# Across population genomic prediction scenarios in which Bayesian variable selection outperforms GBLUP

**DOI:** 10.1186/s12863-015-0305-x

**Published:** 2015-12-23

**Authors:** S. van den Berg, M. P. L. Calus, T. H. E. Meuwissen, Y. C. J. Wientjes

**Affiliations:** Animal Breeding and Genomics Centre, Wageningen University, 6700 AH Wageningen, The Netherlands; Animal Breeding and Genomics Centre, Wageningen UR Livestock Research, 6700 AH Wageningen, The Netherlands; Department of Animal and Aquacultural Sciences, Norwegian University of Life Sciences, P. O. Box 5003, 1432 Ås, Norway

**Keywords:** Genomic prediction, Across population, Bayesian variable selection, GBLUP, Accuracy, Number of independent chromosome segments

## Abstract

**Background:**

The use of information across populations is an attractive approach to increase the accuracy of genomic prediction for numerically small populations. However, accuracies of across population genomic prediction, in which reference and selection individuals are from different populations, are currently disappointing. It has been shown for within population genomic prediction that Bayesian variable selection models outperform GBLUP models when the number of QTL underlying the trait is low. Therefore, our objective was to identify across population genomic prediction scenarios in which Bayesian variable selection models outperform GBLUP in terms of prediction accuracy. In this study, high density genotype information of 1033 Holstein Friesian, 105 Groningen White Headed, and 147 Meuse-Rhine-Yssel cows were used. Phenotypes were simulated using two changing variables: (1) the number of QTL underlying the trait (3000, 300, 30, 3), and (2) the correlation between allele substitution effects of QTL across populations, i.e. the genetic correlation of the simulated trait between the populations (1.0, 0.8, 0.4).

**Results:**

The accuracy obtained by the Bayesian variable selection model was depending on the number of QTL underlying the trait, with a higher accuracy when the number of QTL was lower. This trend was more pronounced for across population genomic prediction than for within population genomic prediction. It was shown that Bayesian variable selection models have an advantage over GBLUP when the number of QTL underlying the simulated trait was small. This advantage disappeared when the number of QTL underlying the simulated trait was large. The point where the accuracy of Bayesian variable selection and GBLUP became similar was approximately the point where the number of QTL was equal to the number of independent chromosome segments (*M*_*e*_) across the populations.

**Conclusion:**

Bayesian variable selection models outperform GBLUP when the number of QTL underlying the trait is smaller than *M*_*e*_. Across populations, *M*_e_ is considerably larger than within populations. So, it is more likely to find a number of QTL underlying a trait smaller than *M*_e_ across populations than within population. Therefore Bayesian variable selection models can help to improve the accuracy of across population genomic prediction.

## Background

In genomic prediction, a reference population consisting of animals with known phenotypes and marker genotypes is used to build a prediction equation to predict genomic estimated breeding values (GEBVs) for selection candidates with an unknown phenotype and a known genotype [1, 2]. The prediction equation contains estimated effects for single nucleotide polymorphism (SNP) markers that are linked to quantitative trait loci (QTL) underlying a trait. The accuracy of estimating GEBVs depends on several factors, such as: the size of the reference population [3–5], the heritability of the trait [6, 7], the level of linkage disequilibrium (LD) between SNPs and QTL [1, 8], and the additive genetic relationships between reference individuals and selection candidates [9, 10].

In numerically small populations, e.g. lines or breeds with a low number of individuals, the size of the reference population is limited which restricts the potential accuracy of genomic prediction [5]. An attractive approach to increase the size of the reference population for a numerically small population is to add individuals from other populations, known as multi population genomic prediction. Simulation studies have indeed shown that the accuracies of genomic prediction can be increased by adding individuals from other populations to the reference population [6]. However, several empirical studies showed that adding individuals from other populations to a reference population from a numerically small population did not result in a significant increase in accuracy compared to within population genomic prediction [11–15]. This low increase in accuracy might be a result of the differences in LD [16–18], allele frequencies and allele substitution effects [19, 20] across populations. Those differences, as well as the absence of close family relationships across populations [21], restrict the accuracy of multi population genomic prediction.

Another factor that is influencing the accuracy of genomic prediction, is the breeding value estimation model. The currently used models can roughly be divided in two groups; models based on genomic best linear unbiased prediction (GBLUP) and nonlinear Bayesian variable selection models [22, 23]. These models differ in their assumption about the distribution of the SNP variances. The original GBLUP model [1] assumes a homogeneous variance among SNPs, i.e. each SNP contributes equally to the total SNP variance. A Bayesian variable selection model assumes heterogeneous variances among SNPs, i.e. some SNPs have a large contribution to the variance and some SNPs have a small or zero contribution. Please note that it is possible to modify the GBLUP model to account for heterogeneous variances as well, as shown by Strandén and Garrick [24]. However, this requires prior knowledge about the SNP variances which is not needed in a Bayesian variable selection model.

The difference in accuracy between GBLUP and a Bayesian variable selection model is dependent on the genetic architecture underlying the investigated trait and genomic properties of the investigated populations. A study that compared the accuracy of within population genomic prediction obtained by a Bayesian variable selection model with accuracies obtained by a GBLUP model, has shown that Bayesian approaches have an advantage over GBLUP when the number of QTL is smaller than the number of independent chromosome segments (*M*_*e*_) in the population [23]. However, when the number of QTL was equal or larger than *M*_*e*_, the accuracy of both statistical methods became equal or, in some cases, GBLUP outperformed the Bayesian variable selection model [23]. To our knowledge, to date the difference in accuracy between a Bayesian variable selection model and a GBLUP model in relation to *M*_*e*_ has not been evaluated for across population genomic prediction, in which reference and selection individuals are from different populations. Our hypothesis is that also in across population genomic prediction, a Bayesian variable selection model will obtain a higher accuracy than GBLUP when the actual number of QTL is smaller than *M*_*e*_ across populations, and the same accuracy as GBLUP when the number of QTL is larger than *M*_*e*_. Wientjes et al. [10] reported that *M*_*e*_ is substantially larger across populations than within a population. Therefore it is more likely that the actual number of QTL underlying a trait is smaller than *M*_*e*_ across populations than within populations.

The objective of this study was to identify across population genomic prediction scenarios in which Bayesian variable selection models outperform GBLUP in terms of prediction accuracy. The accuracies of the Bayesian variable selection model are described in this study using high density genotype information of three dairy cattle breeds. The GBLUP accuracies are presented by Wientjes et al. [25] and are estimated using the same dataset. The phenotypes were simulated such that the underlying factors potentially acting on the accuracy of across population genomic prediction were known.

## Methods

### Data

The dataset used in this study was retrieved from previous research of Wientjes et al. [25], containing the genotypes of 1285 Dutch dairy cows. The cows originated from three different breeds; 1033 Holstein Friesian (HF), 105 Groningen White Headed (GWH) and 147 Meuse Rhine Yssel (MRY) cattle. Each of the individuals originated for at least 87.5 % from one of the three breeds and, therefore, all individuals were considered to be pure-bred animals. For all MRY and GWH animals, nose swabs were used for DNA collection. Nose swabs were collected in accordance with the guidelines for the care and use of animals as approved by the ethical committee on animal experiments of ID-LELYSTAD (protocol: 2011062), and the collection was in accordance with the Dutch Law on Animal Experiments. Before collecting the nose swabs, consent was obtained from the cattle owners. The genotypes from HF animals were obtained from an existing database, and therefore, no approval of an ethical committee was obtained.

The HF individuals were genotyped with the Illumina BovineSNP50 Beadchip (50 k, Illumina, San Diego, CA). The genotypes were imputed to high-density (777 k) using a reference population of 3150 HF individuals by Pryce et al. [26]. The GWH and MRY individuals were genotyped with the Illumina BovineHD Beadchip (777 k, Illumina, San Diego, CA). To increase the power of the analyses, only the SNPs on *Bos Taurus* chromosome (BTA) 13, 23, and 28 were considered. Those three chromosomes form a good representation of the *Bos Taurus* genome, since the LD pattern of BTA 13, 23 and 28 is comparable to the LD pattern of the entire genome [27, 28]. This selection step reduced the total number of SNPs, while it was still possible to benefit from the higher consistency in LD across populations obtained with the 777 k chip compared to the 50 k chip. Non-segregating SNPs from the whole dataset were deleted, i.e. SNPs with a minor allele frequency equal to or lower than 0.5 %. After the quality control and SNP editing, a total of 31,503 SNPs remained. More details on the genotypes, quality control and editing of the SNP data are described in Wientjes et al. [25].

Phenotypes were simulated for different scenarios using two changing variables [25]: (1) the number of QTL underlying the trait, and (2) the correlation between allele substitution effects of the QTL across populations, which represents the genetic correlation between populations [29]. From all 31,503 SNPs in the dataset, 5000 SNPs were randomly selected as candidate QTL. From these 5000 candidate QTL, 3000, 300, 30 or 3 QTL were randomly selected, regardless of the chromosome and allele frequency, to have an effect on the simulated trait. The allele substitution effects of the QTL were sampled from a multi-normal distribution, assuming a genetic correlation of 1.0, 0.8 or 0.4 across all combinations of the three breeds. The remaining 26,503 (31,503-5000) SNPs were used as the group of markers for all analyses.

Simulated phenotypes were calculated as the sum of the true breeding values (**TBV**) and an environmental effect. The TBV for each individual was calculated by multiplying the QTL genotypes with the corresponding allele substitution effects assuming an additive model [25]:

$$ TB{V}_{ij}={\displaystyle {\sum}_{k=1}^m{X}_{ijk}{\alpha}_{jk},} $$

where *TBV*_*ij*_ is the TBV for individual *i* from population *j*, *m* is the number of QTL, *X*_*ijk*_ is the genotype for individual *i* from population *j* at QTL *k*, and *α*_*jk*_ is the true allele substitution effect of QTL *k* in population *j*. The environmental effect was sampled from a normal distribution with a mean of zero and a variance equal to $\left(\frac{1}{h^2}-1\right)$ * (variance of TBV corrected for mean TBV within population). The simulations of the phenotypes were replicated 100 times for each scenario and for each number of QTL underlying the trait, assuming a heritability of 0.95 resembling the heritability of deregressed proofs of bulls based on daughter information. More details about the simulations of the phenotypes are described in Wientjes et al. [25]. Datasets containing the genotype and phenotype information are available on doi:10.5061/dryad.rq80k.

### Scenarios

The accuracy of genomic prediction was evaluated for five different scenarios. An overview of the scenarios is given in Table 1. The first scenario represents a within population scenario, where HF animals were used as reference population to predict GEBVs for HF selection candidates. Since the reference population and the selection candidates were selected from the same population, a 20-fold cross-validation was used to estimate GEBVs. The cross-validations were performed by randomly dividing the HF population in 20 groups where each group consisted of 51 or 52 individuals. In each cross-validation, one group was used as selection candidates and the other 19 groups were used as reference population. In the other four scenarios, GEBVs were estimated for selection candidates of one population using a reference population of one or two other populations, i.e. applying across population genomic prediction, and no cross-validation was required. In all across population scenarios the HF population was included in the reference population.

**Table 1:** Overview of the different scenarios

	Reference population		Selection candidates	
Scenario	Breed(s)	Number of individuals	Breed	Number of candidates
Base	HF	981–982^a^	HF	51–52^a^
1	HF	1033	GWH	105
2	HF & MRY	1180	GWH	105
3	HF	1033	MRY	147
4	HF & GWH	1138	MRY	147

HF = Holstein Friesian; GWH = Groningen White Headed; MRY = Meuse-Rhine-Yssel; ^a^Genomic prediction is based on a 20-fold cross validation using 20 groups of 51 or 52 selection candidates

### Genomic prediction

#### Bayesian variable selection model

The Bayesian variable selection model used in this study to perform genomic prediction was a Bayesian stochastic search variable selection model (Bayes SSVS) [8, 30]. For this model, the following general equation was applied for *n* individuals and *m* markers:

$$ \mathbf{y}={\mathbf{1}}_n\mu +{\sum}_{j=1}^m{\mathbf{X}}_j{\beta}_j+\mathbf{e}, $$

where **y** is the vector of phenotypic records for all *n* individuals; *μ* is the mean; **1**_n_ is a vector with ones of length *n*; **X**_*j*_ is a vector of indicator variables referring to the genotypes for SNP *j* (*j* = 1..*m*) for all individuals, *β*_j_ is the allele substitution effect associated with SNP *j* and **e** is a vector of residuals. The residuals were assumed to be normally distributed, $\mathbf{e} \sim N\left(0,\mathbf{I}{\sigma}_e^2\right)$ [1, 2].

A uniform prior distribution was assigned to *μ*. The allele substitution effects *β*_*j*_ were assumed to be from a mixture of a normal distributions and an indicator variable *γ* determined from which distribution the allele substitution effects were sampled. The indicator variable reflects whether the SNP can be included in the model with a large effect, *γ* = 1, or with a small effect, *γ* = 0. For *γ* = 1, *β*_*j*_ was sampled from $N\left(0,{\sigma}_{\beta}^2\right)$. For *γ* = 0, *β*_*j*_ was sampled from $N\left(0,\frac{\sigma_{\beta}^2}{100}\right)$, so that it had a very small effect. As such, the prior distribution for each SNP effect was ${\beta}_j\Big|{\gamma}_j,{\sigma}_{\beta}^2\sim \left(1-{\gamma}_j\right)N\left(0,\frac{\sigma_{\beta}^2}{100}\right)+{\gamma}_jN\left(0,{\sigma}_{\beta}^2\right)$, with ${\sigma}_{\beta}^2$ sampled from an inverse chi-square distribution.

The prior distribution of the indicator variable *γ* was a Bernoulli distribution for prior probability 1 − *π*: *γ*_*i*_ ~ *bernoulli*(1 − *π*). Variable 1 − *π* reflects on the proportion of SNPs that have a large effect compared to the total number of SNPs. In this study *1-π* was set to 0.01 for all scenarios. The posterior probability of the indicator variable can be sampled directly from its posterior distribution [30]: $p\left(\gamma =1\Big|{\beta}_j,{\sigma}_j^2,{\gamma}_{-j},u,y\right)\sim Bernoulli\ \left(\frac{pd\left({\beta}_j\Big|{\gamma}_{-j},{\gamma}_j=1\right)\left(1-\pi \right)}{pd\left({\beta}_j\Big|{\gamma}_{-j},\kern0.75em {\gamma}_j=1\right)\left(1-\pi \right)+pd\left({\beta}_j\Big|{\gamma}_{-j},\ {\gamma}_j=0\right)\pi}\right)$; where *pd* denotes probability densities, *γ*_*j*_ is the indicator variable and *γ*_*–j*_ refers to all indicator variables except *γ*_*j*_.

A Monte Carlo Markov Chain (MCMC) algorithm implemented using right-hand-side updating [31] was used to perform the analyses. For each analysis, a Gibbs sampling chain with 5000 iterations was run. The first 1000 iterations were discarded as burn-in. For the first replicate of each scenario initially a Gibbs sampling chain of 100,000 iterations with 20,000 iterations as burn-in was run. The GEBVs obtained with 100,000 iterations had a correlation larger than 0.99 with the GEBVs obtained with 5000 iterations. Therefore, a Gibbs sampling chain with 5000 iterations was considered sufficient.

### GBLUP

The GBLUP type of model used to perform genomic prediction was a genomic-relatedness-matrix residual maximum likelihood (GREML) model, run in ASReml [32]. In this model, variances and breeding values are estimated simultaneously using REML, instead of assuming that variances are known, as is the case in a GBLUP model. The GREML analyses were described by Wientjes et al. [25], using the following model equation:

$$ \mathbf{y}=\mathbf{X}\mathbf{b}+\mathbf{Zg}+\mathbf{e}, $$

where **b** is a vector with a fixed breed effect, **X** is an incidence matrix that allocates the fixed breed effect to the individuals, **g** is a vector with genomic breeding values $\left(\mathbf{g}\sim N\left(0,\mathbf{G}{\sigma}_a^2\right)\right)$, **Z** is an incidence matrix that allocates genomic breeding values to the individuals, **G** is a genomic relationship matrix, and ${\sigma}_a^2$ is the additive genetic variance.

### Accuracy of genomic prediction

For both models, the accuracy of genomic prediction was calculated as the Pearson correlation coefficient between the GEBV and TBV across all selection candidates per replicate, since the TBV was known for all selection candidates. Average accuracies and corresponding standard errors were calculated across all replicates of the same scenario. The average accuracies were used for further analyses and comparisons.

### Model comparison

For each of the scenarios, the average accuracy of genomic prediction obtained by the Bayesian variable selection model was compared with the average accuracy obtained by the GBLUP model. It was investigated whether also for across population genomic prediction the accuracies of both models were equivalent when the number of QTL is equal to or higher than *M*_*e*_, as was described for within population genomic prediction [23]. *M*_*e*_ is a statistical concept and represents the number of independent chromosome segments segregating in a population. Due to LD between markers, markers are not segregating independently. The stronger the LD between the markers, the lower the value for *M*_*e*_. Across populations, differences in LD pattern exist, therefore, the number of independent chromosome segment is likely to be higher across populations that within a population. Within a population, *M*_*e*_ can be calculated as [33]:

$$ {M}_e=\frac{1}{Var\left({\mathbf{G}}_{ij}-{\mathbf{A}}_{ij}\right)}, $$

where **G**_*ij*_ and **A**_*ij*_ are respectively the genomic and pedigree relationships between individual *i* and *j*, and the variance is taken across all pairs *ij*. In analogy to this equation, *M*_*e*_ across populations used in this study was calculated by Wientjes et al. [25], as:

$$ {M}_e=\frac{1}{Var\left({\mathbf{G}}_{Pop{.1}_i, Pop{.2}_j}-{\mathbf{A}}_{Pop{.1}_i, Pop{.2}_j}\right)}, $$

where ${\mathbf{G}}_{Pop{.1}_i, Pop{.2}_j}$ and ${\mathbf{A}}_{Pop{.1}_i, Pop{.2}_j}$ are respectively the genomic and pedigree relationships between individual *i* from population 1 and individual *j* from population 2, with the variance taken across all pairs of individuals from population 1 and 2. When two populations were combined in the reference population, the complete reference population was considered as one population in the calculation of *M*_*e*_. An overview of the estimates of *M*_*e*_ is given in Table 2.

**Table 2:** The number of independent chromosome segments (*M*_*e*_) for each scenario

	Scenario		*M*_*e*_^a^	
	Base		185	
	1		1809	
	2		1891	
	3		2435	
	4		2462	

^a^ *M*_*e*_ is estimated by Wientjes et al. [25] as: ${M}_e=\frac{1}{Var\left({\mathbf{G}}_{Pop{.1}_i, Pop{.2}_j}-{\mathbf{A}}_{Pop{.1}_i, Pop{.2}_j}\right)}$; where ${\mathbf{G}}_{Pop{.1}_i, Pop{.2}_j}$ refers to the genomic relationship between individual *i* from population 1 and individual *j* from population 2, ${\mathbf{A}}_{Pop{.1}_i, Pop{.2}_j}$ refers to the pedigree relationship between individual *i* from population 1 and individual *j* from population 2, and the variance is taken over all pair-wise relationships between the individuals in the reference population and the selection candidates

## Results

### Equal allele substitution effects across populations

**Fig. 1:**
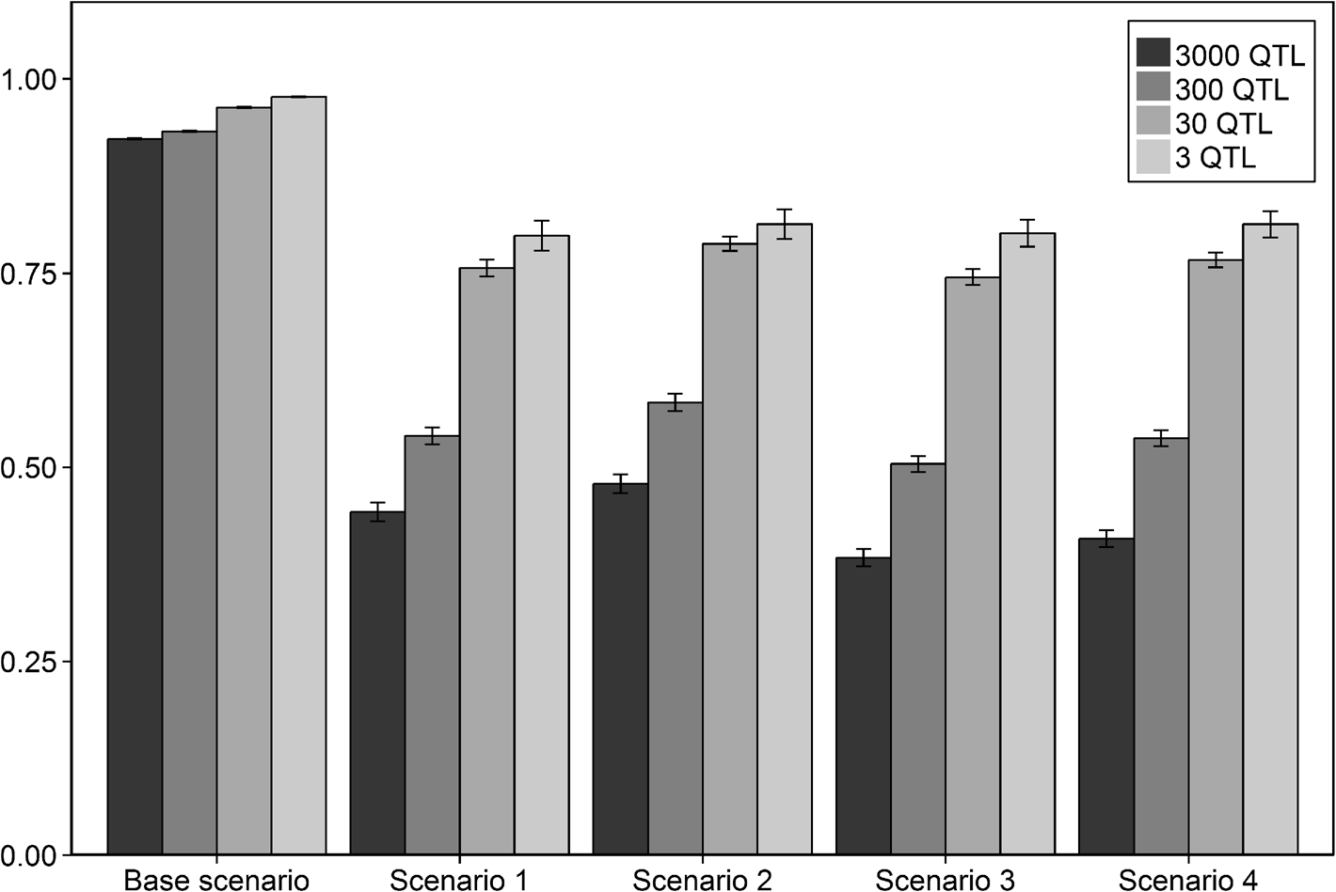
Accuracies of genomic prediction assuming equal allele substitution effects across populations. Mean accuracies of genomic prediction (± standard error) obtained by the Bayesian variable selection model assuming equal allele substitution effects across the three populations for five different scenarios; Base scenario: reference = HF, selection candidates = HF; Scenario 1: reference = HF, selection candidates = GWH; Scenario 2: reference = HF & MRY, selection candidates = GWH; Scenario 3: reference = HF, selection candidates = MRY; Scenario 4: reference = HF & GWH, selection candidates = MRY

The accuracies of genomic prediction obtained with Bayesian variable selection model are shown in Fig. 1 for all scenarios assuming equal allele substitution effects across the three populations. The accuracy of the base scenario, which refers to within population genomic prediction, was high (>0.92) and increased slightly when the number of QTL reduced. The standard errors were very small for the base scenario. Accuracies of the other four scenarios, in which across population genomic prediction was applied, were lower than the accuracies for the base scenario. Standard errors for the across population scenarios were low as well and ranged from 0.009 to 0.02. The accuracy decreased significantly when the number of QTL was increasing. The effect of changing the number of QTL was much stronger for the across populations scenarios than for the within population scenario and the difference between 30 and 3 QTL was much smaller than the difference between 3000 and 300 QTL. The largest difference in accuracy was observed between 300 and 30 QTL underlying the trait. Altogether, our results show that there is an effect of the number of QTL on the accuracy of across population genomic prediction using a Bayesian variable selection model.

Generally, the numerical accuracy was slightly higher for selection candidates originating from the GWH population than for those originating from the MRY population. For both breeds, the accuracies somewhat increased when the other breed was added to the HF reference population.

### Different genetic correlation between populations

**Fig. 2:**
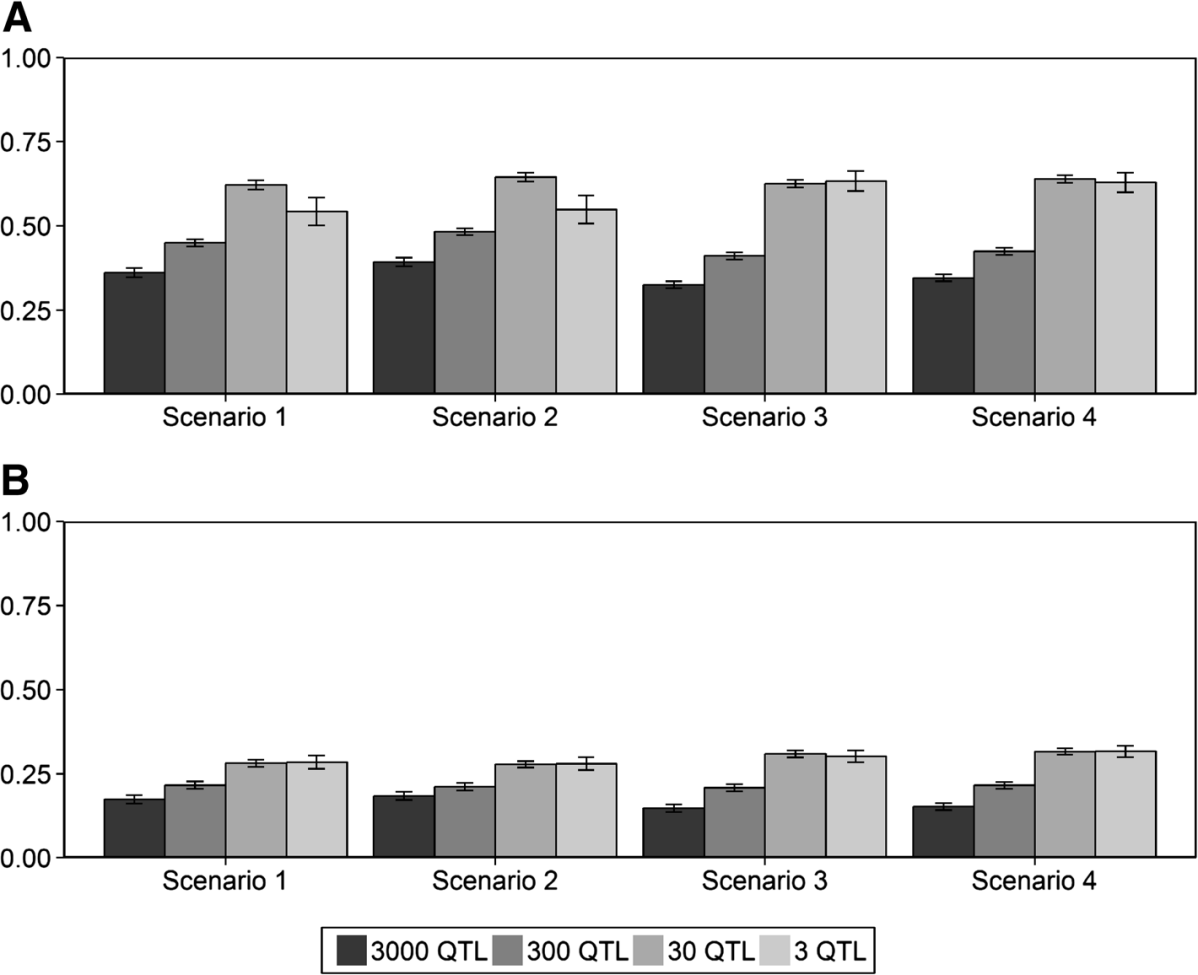
Accuracies of genomic prediction assuming different allele substitution effects across populations. Mean accuracies of genomic prediction (± standard error) obtained by the Bayesian variable selection model assuming genetic correlations of **a** 0.8 or **b** 0.4 across the three populations for four different scenarios; Scenario 1: reference = HF, selection candidates = GWH; Scenario 2: reference = HF & MRY, selection candidates = GWH; Scenario 3: reference = HF, selection candidates = MRY; Scenario 4: reference = HF & GWH, selection candidates = MRY

The accuracies of genomic prediction are shown in Fig. 2 assuming a genetic correlation between the populations of 0.8 (A.) or 0.4 (B.). The standard errors ranged from 0.01 to 0.05 for all scenarios. When there were 3 QTL underlying the simulated trait, the standard errors were larger than when there were 30, 300 or 3000 QTL underlying the simulated trait. Compared to the scenarios with equal allele substitution effects across populations, the accuracy of the scenarios with different allele substitution effects across populations decreased proportional to the correlation in allele substitution effects, i.e. the genetic correlation. So, when the genetic correlation was 0.8, the accuracy was approximately 80 % of accuracy obtained with a genetic correlation between populations of 1, and when the genetic correlation was 0.4, the accuracy was approximately 40 % of the accuracy obtained with genetic correlation between populations of 1.

The effect of the number of QTL on the accuracy was the same for the scenarios that use a genetic correlation different from 1 between populations as for the scenarios with a genetic correlation of 1; the accuracy was increasing when the number of QTL underlying the trait was decreasing. Remarkably, the accuracies for the scenario using GWH as selection candidates (scenario 1 and 2) with 3 simulated QTL was smaller than the accuracies for the scenario that used 30 QTL when the genetic correlation was 0.8.

Again, the numerical accuracies for the selection candidates originating from the breed GWH were generally slightly higher than the accuracies for the selection candidates from the breed MRY. In all scenarios, adding another breed to the HF reference population resulted in a slightly higher accuracy of genomic prediction for the selection candidates.

### Comparison with GBLUP

**Fig. 3:**
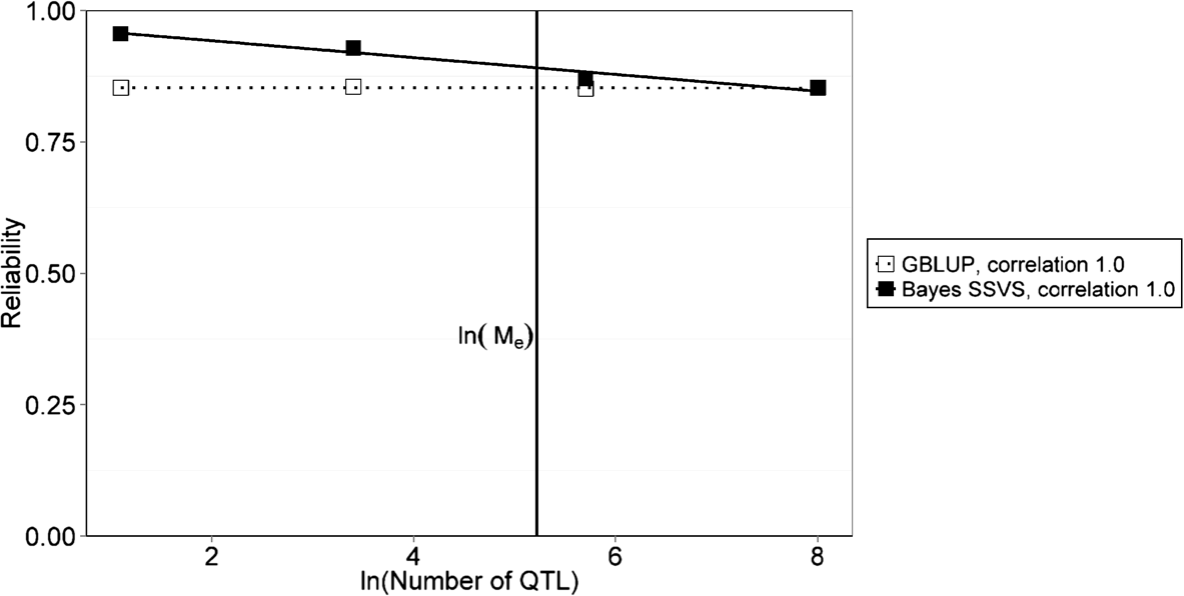
Comparison of the reliability of within population genomic prediction using Bayesian variable selection or GBLUP models. Comparison of the mean reliability of genomic prediction using Bayesian variable selection or GBLUP models for the within population scenario. The vertical line indicates the natural logarithm of the number of independent chromosomes (*M*_*e*_). *M*_*e*_ is estimated by Wientjes et al. [25] as: ${M}_e=\frac{1}{Var\left({\mathbf{G}}_{Pop{.1}_i, Pop{.2}_j}-{\mathbf{A}}_{Pop{.1}_i, Pop{.2}_j}\right)}$; where ${\mathbf{G}}_{Pop{.1}_i, Pop{.2}_j}$ refers to the genomic relationship between individual *i* from population 1 and individual *j* from population 2, ${\mathbf{A}}_{Pop{.1}_i, Pop{.2}_j}$ refers to the pedigree relationship between individual *i* from population 1 and individual *j* from population 2, and the variance is taken over all pair-wise relationships between the individuals in the reference population and the selection candidates

**Fig. 4:**
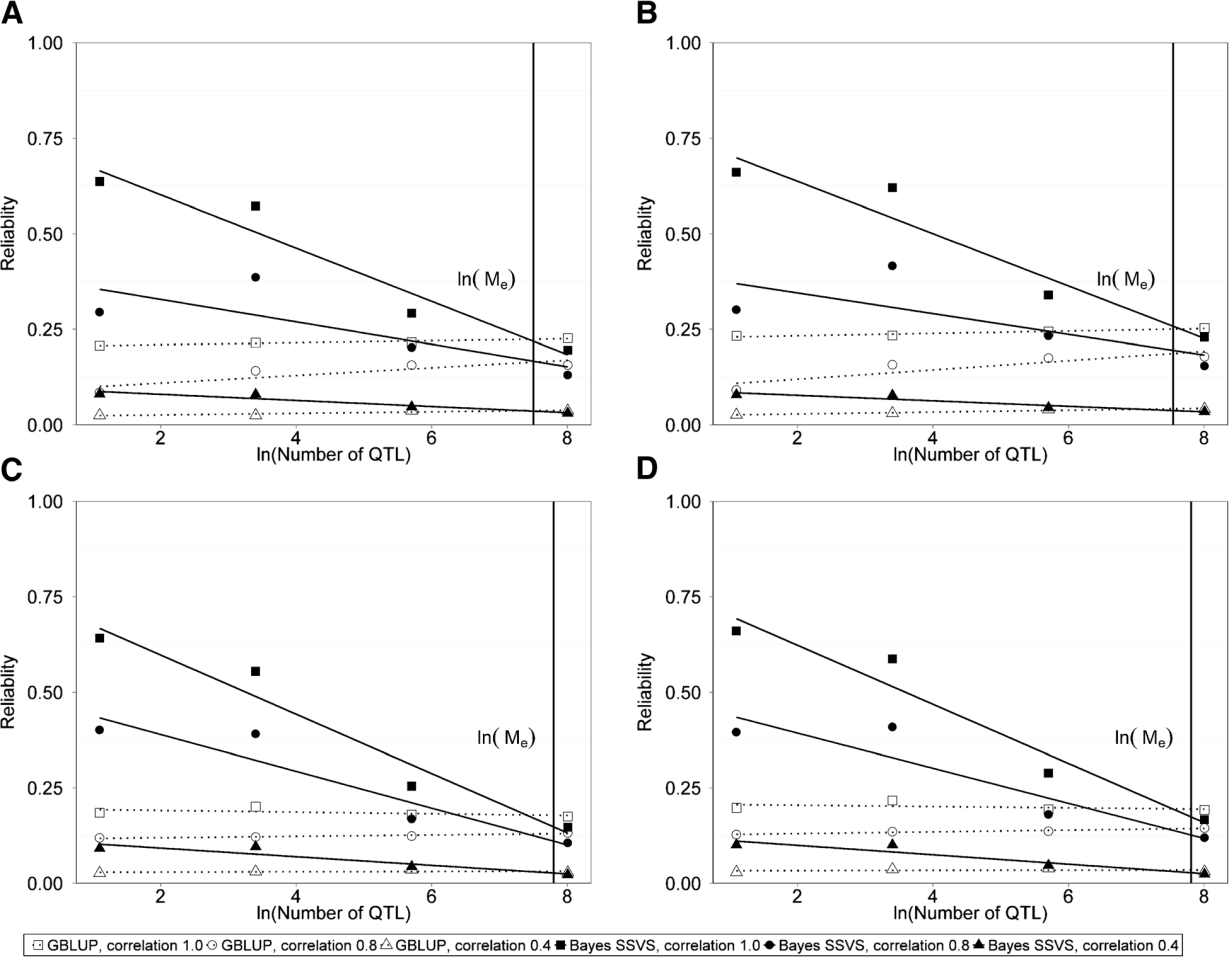
Comparison of the reliability of across population genomic prediction using Bayesian variable selection or GBLUP models. Comparison of the mean reliability of genomic prediction using Bayesian variable selection or GBLUP models for the four across population scenarios with genetic correlation of 1.0, 0.8 or 0.4 across populations; **a** Scenario 1: reference = HF, selection candidates = GWH; **b** Scenario 2: reference = HF & MRY, selection candidates = GWH; **c** Scenario 3: reference = HF, selection candidates = MRY; **d** Scenario 4: reference = HF & GWH, selection candidates = MRY. The vertical line indicates the natural logarithm of the number of independent chromosome segments (*M*_*e*_). *M*_*e*_ is estimated by Wientjes et al. [25] as: ${M}_e=\frac{1}{Var\left({\mathbf{G}}_{Pop{.1}_i, Pop{.2}_j}-{\mathbf{A}}_{Pop{.1}_i, Pop{.2}_j}\right)}$; where ${\mathbf{G}}_{Pop{.1}_i, Pop{.2}_j}$ refers to the genomic relationship between individual *i* from population 1 and individual *j* from population 2, ${\mathbf{A}}_{Pop{.1}_i, Pop{.2}_j}$ refers to the pedigree relationship between individual *i* from population 1 and individual *j* from population 2, and the variance is taken over all pair-wise relationships between the individuals in the reference population and the selection candidates

Figure 3 shows the comparison between the Bayesian variable selection and GBLUP model in relation to the number of QTL for the within population scenario, i.e. the base scenario using HF both as reference population and selection candidates, and Fig. 4 shows the comparison between the Bayesian variable selection and GBLUP model for the across population scenarios, i.e. (a) reference HF, selection candidates GWH, (b) reference HF and MRY, selection candidates GWH, (c) reference HF, selection candidates MRY, (d) reference HF and GWH, selection candidates MRY. Please note that ln(number of QTL) is plotted against the reliability, since the relationship between number of QTL and reliability is approximately linearized by a log-transformation due to the number of QTL occurring in the denominator of the prediction equation of Daetwyler et al. [22].

With a low number of QTL underlying the trait, Bayesian variable selection performed always better than GBLUP. When the number of QTL was higher, the difference between the reliabilities of both approaches became smaller and eventually the Bayesian variable selection model resulted in reliabilities comparable to GBLUP. For the within population scenario (Fig. 3), the difference between the Bayesian variable selection model and GBLUP model was already quite small with 3 QTL underlying the trait, and when the number of QTL was 300, both reliabilities were almost equal to each other. For the across population scenarios (Fig. 4), the difference in reliability of the Bayesian variable selection and GBLUP was much larger, and both reliabilities became equal when approximately 2000 QTL were underlying the trait, which was at a much higher number of QTL than within population. This is in agreement with the estimated values for *M*_*e*_, which were much lower within population than across populations (see Table 2).

## Discussion

### The accuracy of across population genomic prediction

The objective of this study was to identify across population genomic prediction scenarios in which a Bayesian variable selection model outperforms GBLUP in terms of prediction accuracy. The used dataset contained real genotype information of 1033 HF, 147 MRY and 105 GWH animals [25]. Phenotypes of all individuals were simulated with two changing variables: (1) the number of QTL underlying the trait, and (2) the genetic correlation between the populations.

The accuracies for within population genomic prediction were substantially higher than the accuracies for across population genomic prediction for both the Bayesian variable selection model and the GBLUP model. This is in line with the general observation in literature, e.g. [13, 22, 34] and can be explained by the differences between populations, such as differences in LD patterns, allele frequencies and allele substitution effects. These differences in combination with the absence of close family relationships restrict the accuracy of genomic prediction across populations [16, 17, 19–21, 35].

As a result of non-additive effects in combination with different allele frequencies, allele substitution effects might differ across populations [29]. Those differences in allele substitution effects between populations can be summarized in the genetic correlation between the populations. This genetic correlation is shown to be an important factor for the accuracy of across population genomic prediction obtained by GBLUP [25]. A decrease in the genetic correlation resulted in a reduction in accuracy obtained by GBLUP proportional to the genetic correlation. Our results show that the genetic correlation similarly affects the accuracy of across population genomic prediction obtained by a Bayesian variable selection model. This relation between the genetic correlation between populations and the obtained accuracy has also been reported for multi population genomic prediction obtained with a Bayesian variable selection model that was similar to the model used in this study [36].

The genetic correlation between populations was simulated in this study as the correlation between allele substitution effects across populations, indicating that allele substitution effects were different across populations. Another possible reason for a genetic correlation between populations lower than 1 is that different QTL might underlie a trait. The accuracy is influenced by the value of the genetic correlation, as can be seen from the equation to calculate the accuracy of across and multi population genomic prediction [25]. This suggests that the underlying cause of the genetic correlation has no effect on the accuracy of across and multi population genomic prediction. Therefore, we think that simulating a genetic correlation different from 1 in another way would not have influenced the results of this study.

In this study it is demonstrated that the accuracy of across population genomic prediction obtained by a Bayesian variable selection model strongly depends on the number of QTL underlying the simulated trait. It was shown that the Bayesian variable selection model obtained the highest accuracies when the number of QTL underlying the trait was small. When the number of QTL increased, the accuracy obtained by the Bayesian variable selection model declined. When GWH animals were used as selection candidates and the genetic correlation was 0.8, a slightly higher accuracy was obtained when 30 QTL were underlying the trait than when 3 QTL were underlying the trait. This result was also obtained using the same data with a GBLUP model [25] and is probably due to a lower simulated genetic correlation when only 3 QTL were underlying the trait as a result of a larger sampling error on the simulated genetic correlation. For the scenarios with 3 QTL underlying the trait, the average simulated genetic correlation was lower than 0.8 (average correlations ± standard errors were 0.74 ± 0.040 between HF and GWH, 0.75 ± 0.035 between HF and MRY, and 0.77 ± 0.043 between GWH and MRY), since the correlation can only increase till 1, but decrease till −1. When 30 QTL were underlying the trait, the average simulated genetic correlations were between 0.79 and 0.80.

The dependency of the accuracy on the number of QTL in both within and across population genomic prediction using a Bayesian variable selection model was also found in literature [17, 23, 37–39]. For example, Coster et al. [37] investigated the effect of the number of QTL on the accuracy of within population genomic prediction. They found that the accuracy of within population genomic prediction obtained by a Bayesian variable selection model decreased when the number of simulated QTL increased. Chen et al. [36] have found similar results for multi population genomic prediction using a Bayesian variable selection model.

In contrast to the accuracy of genomic prediction obtained by a Bayesian variable selection model, the accuracy obtained by GBLUP appears unaffected by the number of QTL underlying the trait. Therefore, the Bayesian variable selection model was clearly superior to GBLUP for across population genomic prediction when the number of QTL was small, i.e. less than ~2000 QTL in our simulated data. Please note that we have focussed only on three chromosomes, indicating that this is equivalent to ~10 times more QTL when the whole genome of individuals from those breeds was considered. When the number of QTL increased, the difference in accuracy between the Bayesian variable selection model and GBLUP model decreased, until the accuracy of the Bayesian variable selection model was similar to the accuracy obtained with GBLUP. An empirical study applying across population genomic prediction also showed higher accuracies when a Bayesian variable selection model was used compared to a GBLUP model for milk production traits [22], with an average accuracy of 0.30 using a Bayesian variable selection model and of 0.01 using a GBLUP model when Holstein Friesian animals were used to predict Jerseys. Moreover, equal or higher accuracies were obtained with a Bayesian variable selection model than with a GBLUP model for different multi population genomic prediction scenarios using real data, with an average numerical difference of 0.03 between both models [40, 41].

The differences in dependency of the accuracy from different genomic prediction models on the number of QTL can be explained by differences in the model mechanism. The original GBLUP model [1], as well as the GREML model used in this study, assumes an infinitesimal model, i.e. each SNP is assumed to explain an equal small amount of the variation. Bayesian variable selection models make a distinction between the SNPs by allocating a large effect to a small subset of SNPs with a clear association with the trait, while all other SNPs are assumed to have a small effect. When the number of QTL is smaller than *M*_*e*_, there is a clear advantage of selecting a subset of SNPs to allocate large effects since it reduces the number of effects that has to be estimated. When the number of QTL is larger than *M*_*e*_, each SNP appears to have a small effect on the trait and there is no clear set of SNPs that the model can select to have a large effect. Therefore, the number of estimated effects becomes equal to *M*_*e*_ and the advantage of a Bayesian variable selection model over GBLUP diminishes. A posteriori the model assigns a fairly equal amount of variance to each SNP, an approach that is equivalent to the assumption of the infinitesimal model underlying GBLUP.

Daetwyler et al. [23] investigated the difference in factors acting on the accuracy of within population genomic prediction obtained by a Bayesian variable selection model and GBLUP model. They reported that the accuracy of GBLUP is independent from the number of QTL, but is dependent on genomic properties of the population such as the effective population size and LD. The genomic properties of the population can be summarized in the parameter *M*_e,_ the number of independent chromosome segments [23]. *M*_e_ is a statistical concept that links genomic properties of the population to the statistical analysis. It can be derived from the consistency of variation in LD across the genome and the variation in relationship around their expectations between individuals [5]. In a wider sense, *M*_*e*_ can be interpreted as the number of independent markers needed to capture all the variation in QTL effects or the number of independent effects that have to be estimated. Thus the accuracy of genomic prediction obtained by GBLUP is dependent on *M*_*e*_; the higher *M*_*e*_, the more independent effects have to be estimated and the lower the accuracy of genomic prediction. For a Bayesian variable selection model this relationship is slightly more complicated. For within population genomic prediction, it is shown that when the number of QTL is lower than *M*_*e*_, the accuracy obtained by Bayesian variable selection decreases when the number of QTL is increasing [23]. When the number of QTL is larger than *M*_*e*_ and a QTL is located on each of the independent chromosome segments, the accuracy is independent from the number of QTL and similar to the accuracy obtained by GBLUP [23], since the number of estimated effects is equal to *M*_*e*_ in both GBLUP and the Bayesian variable selection model. The results of the within population scenario in our study also show that the difference between GBLUP and Bayesian variable selection declined for a higher number of QTL underlying the trait, as is shown in Fig. 3. In this figure, the lines representing the reliability, i.e. the square of the accuracy, of the Bayesian variable selection and GBLUP model, however, cross at a much higher number of QTL than *M*_e_. This might partly be a result of randomly sampling QTL, allocating no QTL to some independent chromosome segments and more than one QTL to other segments, reducing the number of independent QTL segregating in the population. The crossing point at a higher number of QTL might also be a result of plotting one linear function through the different data points, although a breaking point in the function is expected at *M*_e_. Given the results described in literature, we would expect that the Bayesian variable selection and GBLUP accuracy within population would be approximately equal when the number of independent QTL was higher than *M*_e_ in our study. The results of the across population scenarios in this study show that this principle, described by Daetwyler et al. [23] for within population genomic prediction, is also applicable for across population genomic prediction (Fig. 4). Due to the higher number of chromosome segments across populations, each segment is smaller, and the chance for multiple QTL on the same segment is decreased. Therefore *M*_*e*_ might be a better approximation for the crossing point for across population genomic prediction than for within population genomic prediction when QTL are randomly distributed. For both scenarios, however, *M*_e_ can be considered to be an important parameter.

Wientjes et al. [25] have shown that *M*_e_ is larger across populations than within population. They have reported that estimates for *M*_*e*_ were approximately 10 times larger across populations than within population [25]. The higher estimates for *M*_e_ across populations can be explained by the fact that *M*_e_ is dependent on the level of relatedness between individuals [7, 10]. When individuals are closely related, LD is strong and a lower number of informative markers is needed to explain the variation in QTL effects, indicating a small value for *M*_e_. However, it is known that there is an absence of closely related individuals across populations and individuals differ strongly in LD patterns [16, 21]. So more informative markers are needed to explain the variation in QTL effects and the value for *M*_e_ is higher. Due to the higher value for *M*_e_ across populations, it is more likely to have a number of QTL underlying a trait that is smaller than *M*_e_.

A question that remains is; how many QTL are underlying the important traits for selection? In dairy cattle, it is well known that a large part of the genetic variation in fat content in milk is explained by one gene; DGAT1 (*diacylglycerol O-acyltransferase 1*) [42]. For this trait, it was already shown for within population genomic prediction that Bayesian variable selection models can obtain a higher accuracy compared to GBLUP [4]. Therefore, a substantial benefit of Bayesian variable selection models over GBLUP can be expected for this trait when across or multi population genomic prediction is applied, as is shown by Hayes et al. [22]. For most quantitative traits, a very small number of QTL with large effects has been found [43, 44]. This suggests that a large number of QTL with only small effects are underlying quantitative traits. For those traits, the accuracy of within population genomic prediction was about equal when using a Bayesian variable selection model compared to GBLUP [4, 45]. For across and multi population genomic prediction scenarios, however, higher accuracies were obtained using Bayesian variable selection models for at least some of the traits [12, 13, 22]. This shows that for at least a proportion of the quantitative traits, it can be advisable to use Bayesian variable selection models when across or multi population genomic prediction is applied. A disadvantage of Bayesian variable selection models is, however, its potentially larger computational requirements. Therefore, it might be good to carefully weigh the potential increase in accuracy against the larger requirements to decide on the best model for practical applications.

## Conclusion

The accuracy of across population genomic prediction obtained by a Bayesian variable selection model is dependent on the number of QTL underlying the trait, with the highest accuracy when the number of QTL underlying the trait is small. When the number of QTL underlying the trait is increasing, the accuracy of genomic prediction obtained by a Bayesian variable selection model declines and eventually becomes equal to the accuracy obtained by GBLUP. The point where the accuracy obtained by Bayesian variable selection becomes equivalent to the accuracy obtained by GBLUP can be approximated by the number of independent chromosome segments (*M*_*e*_). So, Bayesian variable selection models have an advantage over GBLUP when the number of QTL is smaller than *M*_*e*_. Across populations *M*_e_ is larger than within populations, indicating that it is more likely to find a number of QTL underlying a trait smaller than *M*_e_ across populations than within populations. Therefore, Bayesian variable selection models can improve the accuracy of across population genomic prediction compared to GBLUP for at least some traits that are influenced by a relatively small number of QTL.

### Availability of supporting data

Data are available on the Dryad Digital Repository: doi:10.5061/dryad.rq80k.

## Abbreviations

SNP: Single nucleotide polymorphism
QTL: Quantitative trait loci
GEBVs: Genomic estimated breeding values
LD: Linkage disequilibrium
GBLUP: Genomic best linear unbiased prediction
*M*_*e*_: number of independent chromosome segments
HF: Holstein Friesian
GWH: Groningen White Headed
MRY: Meuse-Rhine-Yssel
BTA: *Bos Taurus* chromosome
TBV: True breeding value
Bayes SSVS: Bayesian stochastic search variable selection
